# Screening the thermophilic and hyperthermophilic bacterial population of three Iranian hot-springs to detect the thermostable α-amylase producing strain

**Published:** 2010-03

**Authors:** J Fooladi, A Sajjadian

**Affiliations:** Department of Biology, Faculty of Sciences, Alzahra University, Vanak, Tehran, Iran

**Keywords:** Thermostable enzyme, α- amylase, Thermophilic Bacterium, Iran

## Abstract

**Background:**

Screening is a routine procedure for isolation of microorganisms which are able to produce special metabolites. Purified thermostable α-amylase from bacterial sources is widely used in different industries. In this study we analyzed samples collected from three different hot springs in Iran to detect any strains capable of producing thermostable α-amylase.

**Materials and Methods:**

Hot water samples from Larijan (67°C, pH 6.5), Mahallat (46°C, pH 7), and Meshkinshahr (82°C, pH 6), were cultivated in screening starch agar plates and incubated at 65°C for 24 hours. Thereafter, the plates were stained with Gram's iodine solution.

**Results and Discussion:**

The bacterial colonies from the Meshkinshahr hot-spring produced the largest haloforming zone. Based on the phenotypic tests, the strain was identified as *Bacillus sp*. The culture condition was optimized for biosynthesis of α-amylase. The enzyme was produced at maximum level when it was incubated at 70°C in the presence of soluble starch (1%) at pH 6. The addition of calcium (10 mM) and peptone (1%) to the mineral medium, shortened the lag period and improved the growth and α-amylase synthesis. The addition of glucose (1%) to the culture greatly diminished the syntheses of α -amylase. Importantly, the enzyme extract retained 100% activity when incubated for 45 minutes at 100°C.

**Conclusion:**

The Meshkinshahr hot-spring is rich in the *Bacillus spp* thermostable α-amylase producing strain of the thermophilic bacterial population. Iranian hot-springs like Meshkinshahr, have large microbial storages and can be used as sources of different biological products like enzymes. The enzyme which was produced with *Bacillus sp.* could hydrolyse polymers like starch and was used at laboratory scale successfully.

## INTRODUCTION

α-Amylase (EC3.2.1.1, 1, 4-a-D-glucan glucano-hydrolase, endoamylase) hydrolyzes starch, glycogen, and related polysaccharides by randomly cleaving internal α-1,4-glucosidic linkages (1–4). It is widely produced by various bacteria, fungi, plants, and animals and has a major role in the utilization of polysaccharides. α-Amylase is an important industrial enzyme (4, 5). As well as being used as an additive in detergents, it can be used for such things as the removal of starch spots from textiles, the liquefaction of starch, and the proper formation of dextrin in baking. The thermostability of the α-amylase must be matched to its application (5). For example, thermostable α-amylase is used for the liquefaction of starch in starch industries (6, 7). In the present study we screen and isolate thermophilic and hyperthermophilic bacteria to detect thermostable α-amylase producing bacteria and optimize conditions for better cultivation and production of the enzyme.

## MATERIALS AND METHODS

Culture medium: Medium consisting of peptone (2%), yeast extract (1%), NaCl (1%), and agar (2%) (pH 7) was used for selection of thermophilic bacteria (6–8). To detect starch digesting enzymes from bacterial isolates, a 2nd agar medium (Agar B) containing 1% soluble starch, 0.2% yeast extract, 0.5% peptone, 0.05% MgSO4, 0.05% NaCl, 0.015% CaCl2 and 2% agar at pH 7.0 (6–8) was formulated.

A broth medium designated as medium C with the following formula was used for enzyme production: (H4)2SO4 (2%), MgSO4.7H2O (0.05%) tryptone (0.5%) yeast extract (0.5%), soluble starch (1%), KH2PO4 (0.03%), K2HPO4 (0.07%) and FeSO4.7H2O (0.001%) (6–8).

**Screening, Isolation, and Identification.** The liquid and sediment samples for screening were obtained from individual hot-springs in Larijan (67°C, pH 6.5), Mahallat (46°C, pH 7), and Meshkinshahr (82°C, pH 6).

At first, the sediment was removed by centrifiugation at 3000g for 10 minutes, and cultivated in liquid medium A. This was incubated at 65°C on a rotary shaker (150 rpm) for 48 hours. Thereafter culture suspensions in medium A were spread on solid medium A. The plates were incubated at 65°C for 48 hrs to select thermophilic bacteria. The bacterial colonies appearing on plate A were transferred to medium B. These cultures were incubated at 65°C for 48 hours. Amylase producing colonies were selected by flooding the media B plates with iodine solution. Microbial properties of the isolated strain were determined according to the methods described in Bergey‘s Manual of Systematic Bacteriology (9).

**Enzyme production medium.** Initially, the bacterial strain was grown in liquid medium C for 48 hours at 65°C as preculture. Subsequently the effects of varying pH values (5–10) and temperatures (40–80°C) on production of α-amylase by the bacterium was also investigated. In separate experiments, the medium was supplemented with calcium (10mM), peptone (1%), and glucose (1%) to study the effect of these nutrient on growth and enzyme production by this strain.

**α-Amylase assay.** The amylase assay was based on reduction of blue color intensity resulting from enzyme hydrolysis of starch (1). The reaction contained 1 ml enzyme (cell free supernatant) and 10 ml of 1% starch solution incubated at 50°C for 10 min. The reaction was stopped by adding 10 ml of 0.1N HCl. One millilitre of this acidified solution was added to 10 ml of 0.1N HCl. 1 ml of this was added to 10 ml iodine solution (0.05% iodine in 0. 5% KI). The optical density of the blue-colored solution was determined at 660 nm. The same procedure was repeated using 1 ml distilled water instead of the enzyme sample in order to measure the optical density without the enzyme. One unit of enzyme activity (defined unit number) is defined as DUN and the quantity of enzyme that causes 1% reduction of blue color intensity of starch-iodine solution a 50°C in 1 min (6).

**Effect of pH on enzyme activity.** The optimal pH for enzyme activity was determined by changing the assay reaction mixture pH using the following buffers (0.1 M): sodium acetate (pH 5.0), sodium phosphate (pH 6.0–7.0), Tris–HCl (pH 8), glycine–NaOH buffer (pH 9–10) and 1% soluble starch as substrate. The residual enzyme activity was determined as described earlier (6–8).

**Effect of temperature on enzyme activity.** The optimum temperature for the enzyme activity was evaluated by measuring the α-amylase activity at different temperatures (40–80°C) in 0.1 M sodium phosphate buffer (pH 7.0) and 1% soluble starch. The effect of temperature on amylase stability was determined by measuring the residual activity after 1 hr. and 24 hours incubation at different tempretures (60, 70, 80 and 100°C) (6, 7).

## RESULTS

**Fig. 1:**
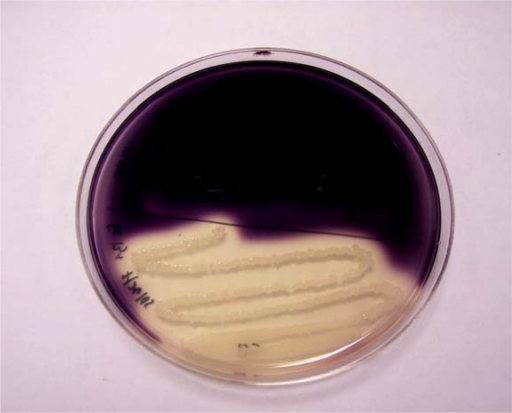
Result of screening in plate B.

We have isolated and successfully cultivated a thermophilic bacterium producing α-amylase. After screening and staining plates with Gram's iodine solution, it was observed that colonies of the isolated bacterial strain from the Meshkinshahr hot-spring generate the largest haloforming zone (Fig. 1). The strain was a facultative anaerobic Gram-positive bacillus, with polar spores. The strain possessed the ability to hydrolyse starch. It was. Catalase was positive. Indole was not formed, and Nitrates were reduced to nitrites. The final pH after growth in nutrient broth was about 6. The strain grew in nutrient broth at 37°C to 80°C with growth temperature being optimum at 70°C for 24 hrs. The strain was identified as *Bacillus* sp by the criteria of Bergey's Manual of Systematic Bacteriology (9).

**Fig. 2:**
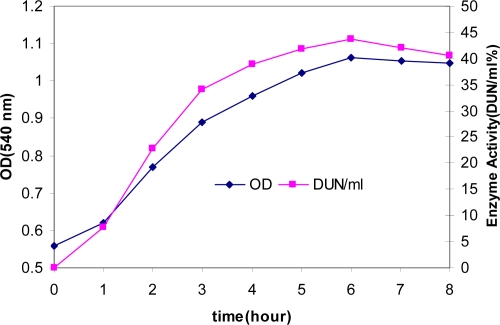
Time-course on growth and α-amylase production.

**Fig. 3:**
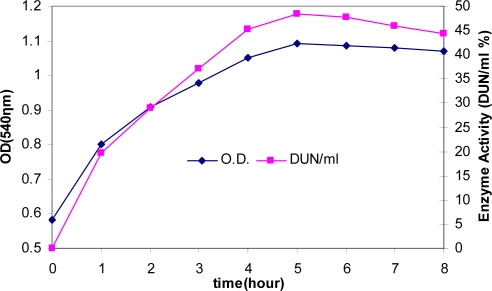
Effect of 10 mM calcium, 1% peptone and 0.5% yeast extract on growth and α-amylase production

**Enzyme production.** Measurements of enzyme activity and growth of *Bacillus* sp at time intervals are shown in Fig. 2. Initially, the organism was grown in liquid medium (Fig. 2). Cell growth was measured at 540 nm (6)-later in liquid medium supplemented with calcium (10 mM) (Fig. 3). The addition of 10 mM calcium and peptone (1%) to the liquid medium improved growth and amylase production. Since the enzyme is known to be a calcium metalloenzyme, it is possible that the results are due to greater availability of calcium ions.

**Fig. 4:**
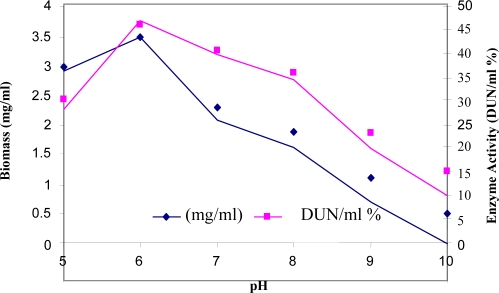
pH Effect on growth and α-amylase production.

The organism showed poor growth in culture media adjusted to pH 9, and 10 (Fig. 4). There was stimulation of enzyme synthesis with an increase in pH from 5 to 8 and the a results of enhanced bacterial growth.

**Fig. 5:**
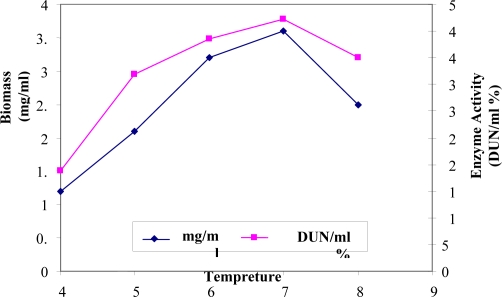
Effect of tempreture on growth and α-amylase production.

Enzyme synthesis occurred at temperatures between 40 and 80°C. The bacterium could grow satisfactorily at all temperatures tested but the maximum α-amylase activity was achieved at 70°C (Fig. 5). A reduction in enzyme activity was observed at temperatures above 70°C.

**Fig. 6:**
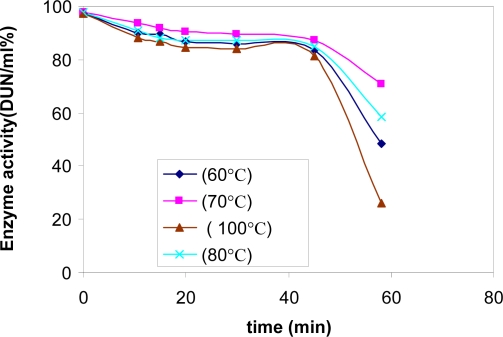
Stability of thermophilic α-amylase at different tempreture and different times.

**Characterization of crude α-amylase.** The effect of pH on α-amylase activity of this bacterial strain was found in very broad pH range. The optimum pH was found to be 6. The optimum temperature for enzyme activity was between 60°C and 80°C. A reduction in enzyme activity was observed at values above 80°C. The enzyme extract retained 100% of initial activity when incubated for 45 min at 100°C respectively (Fig. 6). After this time the activity decreased drastically.

**Fig. 7:**
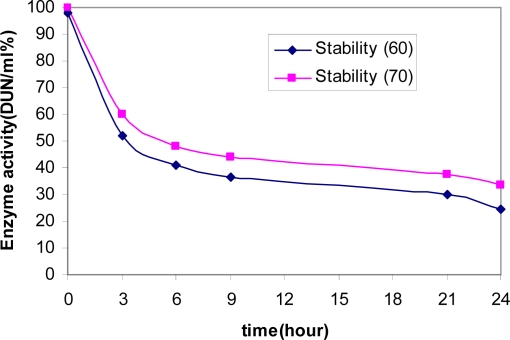
Effect of temperature stability of α-amylase.

After 24 hours at 70°C, and 60°C, the enzyme retained 41% of its initial activity (Fig. 7) After 45 minute, the activity decreased drastically.

## DISCUSSION

**Screening and Optimization of the condition of the medium.** The results of screening and staining Agar B plates with Gram's iodine solution showed that colonies of isolated bacterial strain from the Meshkinshahr hot-spring has the largest haloforming zone. The strain was a Gram-positive facultative anaerobic bacillus with polar spores. It possessed the ability to hydrolyse starch and was catalase positive. Indole was not formed, and nitrates were reduced to nitrites. The final pH after growth in nutrient broth was about 6. The strain grew in nutrient broth at a temperature range of 37°C to 80°C with an optimum at 70°C for 24 h. From these results, the strain was identified as *Bacillus* sp by the criteria of Bergey's Manual of Systematic Bacteriology (9).

The addition of 10 mM calcium and peptone (1%) to the liquid medium improved the growth and amylase production. Since the enzyme is known to be a calcium metalloenzyme, it is possible that the results are due to greater availability of calcium ion. These results are similar to the findings of Hewitt and Solomons with cultures of *Bacillus amyloliquefaciens* (6). Amylase synthesis by several microorganisms has been correlated to the presence or absence of various amino acids and complex nitrogenous sources in the culture medium (2, 4) (6–8, 10). Indeed, the peptone to the liquid medium shortened the lag period and increased both the dry weight of the cell and enzyme synthesis. Therefore, results suggest yeast extract and peptone is favored for the growth and synthesis of amylase by the organism studied (6).

Among the physical parameters, the pH of the growth medium plays an important role by inducing morphological change in the organism and in enzyme secretion. Most of the *Bacillus* strains used commercially for the production of α-amylases have an optimum pH between 6.0 and 9.0 for growth and enzyme production (6). In our study the *Bacillus* strain showed optimum growth and maximum αamylase yield at pH 6.

The influence of temperature on amylase production is related to the growth of the organism (3, 4, 10, 11). A wide range of temperature (35–80°C) have been reported for optimum growth and α-amylase production in bacteria (6). Very recently Konsula and Liakopoulou-Kyriakides (2004) reported that a thermophilic *B.subtilis* strain, isolated from fresh sheep's milk, produced maximum extracellular thermostable α-amylase at 40°C in a medium containing low starch concentration (6).

Optimum activity at pH values as low as 3.5 or as high as 12 has been reported (6).

The optimum temperature for enzyme activity was about 70°C. A reduction in enzyme activity was observed at values above 80°C. According to the results presented, (tempreture: 70°C, pH 6 and in the present of 1% petone and Ca^2+^).

In conclusion, we isolated *Bacillus* spp thermostable α-amylase producing strain. The enzyme produced with this *Bacillus sp.* could hydrolyse polymers like starch and be used in laboratory scale successfully. Iranian hot-springs, like the one at Meshkinshahr, have large amount of microbian storages and can be used as a source for different biological products like enzymes.
